# Primary and recurrent ovarian high-grade serous carcinomas display similar microRNA expression patterns relative to those of normal ovarian tissue

**DOI:** 10.18632/oncotarget.12045

**Published:** 2016-09-15

**Authors:** Eun Ji Nam, Sunghoon Kim, Taek Sang Lee, Hee Jung Kim, Jung Yun Lee, Sang Wun Kim, Jae Hoon Kim, Young Tae Kim

**Affiliations:** ^1^ Institute of Women's Life Medical Science, Women's Cancer Clinic, Department of Obstetrics and Gynecology, Yonsei University College of Medicine, Seoul, Korea; ^2^ Department of Obstetrics and Gynecology, SMG-SNU Boramae Medical Center, Seoul, Korea

**Keywords:** recurrence, ovarian cancer, microRNA, chemoresistant

## Abstract

Most patients with epithelial ovarian cancer eventually die due to recurrence. However, little is known about the microRNA (miRNA) expression pattern and its involvement in recurrent ovarian cancer. We analyzed miRNA expression profiles related to the recurrence of advanced ovarian high-grade serous carcinoma (HGSC) using miRNA microarrays. Between May 2006 and December 2012, 37 ovarian HGSC patients underwent secondary cyto-debulking surgery at recurrence. Among them, only 8 pairs of primary and recurrent tumor samples were deemed to be adequate for analysis. The expression profiles of primary ovarian HGSC compared with normal ovarian tissue were significantly consistent with those of recurrent ovarian HGSC compared with normal ovarian tissue (correlation coefficient = 0.81, *P* = 0.0078). Among 31 miRNAs that increased by more than 4-fold in primary tumors compared with normal ovarian tissue, 27 were also significantly increased in recurrent tumor samples. Likewise, among 35 miRNAs that decreased by more than 4-fold in primary tumors compared with normal ovarian tissue, 34 were also significantly decreased in recurrent tumor samples. We identified 60 miRNAs that were significantly increased in recurrent serous ovarian carcinoma compared with primary tumor tissue, including *miR-630*, *miR-370*, and *miR-575*. Additionally, 52 miRNAs were significantly decreased in recurrent samples, including *miR-509-3p*, *miR-514a-3p*, and *miR-506-3p*. Our results demonstrate that primary and recurrent ovarian HGSC displayed similar miRNA expression patterns. Nevertheless, altered miRNA expression could be implicated in the recurrence of ovarian HGSC, and further study is needed to validate these data in independent cases using a homogeneous methodology.

## INTRODUCTION

Epithelial ovarian cancer is the most lethal malignancy among gynecological cancers, reflecting early peritoneal dissemination with advanced-stage disease at diagnosis [1]. The incidence of ovarian carcinoma exceeded 22,000 new cases in the United States in 2012 and was responsible for approximately 14,000 cancer-related deaths [2]. Although radical surgery and chemotherapy frequently afford complete remission, most patients eventually succumb to chemoresistant disease [1]. Therefore, a greater understanding of the molecular mechanisms related to recurrence is needed for the development of enhanced therapeutic approaches to treat ovarian cancer.

MicroRNAs (miRNAs) are non-coding, single-stranded RNAs of approximately 22 nucleotides in length that constitute a novel class of gene regulators [3]. miRNAs are involved in various biological processes, including cell differentiation, proliferation, apoptosis, carcinogenesis, and metabolism [4]. Although previous studies have characterized gene expression profiles, including those of miRNAs, in primary ovarian cancers [5–7], only limited data are available regarding miRNA gene expression profiles in recurrent ovarian cancers [8–13]. This is largely due to a lack of available tumor specimens from recurrent ovarian cancer patients, since secondary surgeries or biopsies are rarely performed in these patients. In addition, there are no suitable *in vitro* or *in vivo* pre-clinical models to study recurrent cancers.

In this study, we analyzed miRNA expression profiles associated with the recurrence of advanced high-grade serous ovarian carcinoma (HGSC). To minimize the effect of genetic differences among individual patients, we evaluated paired primary ovarian tumor tissue and corresponding tumor tissue from the same patients at recurrence.

## RESULTS

### Comparison of miRNA expression profiles between fresh-frozen and paraffin-embedded ovarian cancer tissue

**Figure 1:**
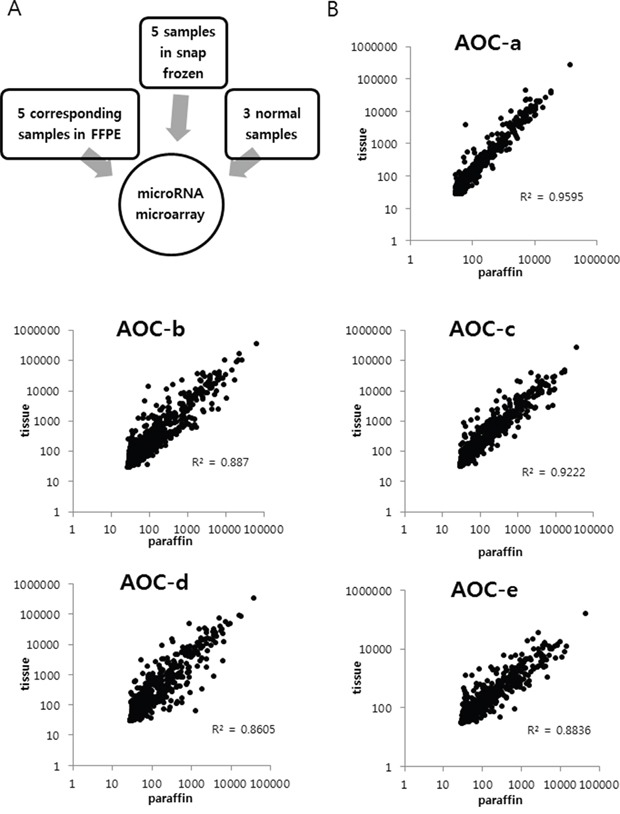
Comparison of miRNA expression between fresh snap-frozen (SF) and paraffin-embedded (FFPE) ovarian cancer tissue All patients had primary advanced serous ovarian cancer. **A.** Five samples in SF tissues and five corresponding FFPE samples from the same patients were compared. **B.** A significant correlation was observed between the miRNA expression profiles of snap-frozen tissue and FFPE tissue in five patients (AOC-a, b, c, d, and e). (*P* < 0.001).

To determine whether miRNA expression profiles are preserved in FFPE specimens compared with those in SF specimens, expression of 1205 human miRNAs in SF and FFPE specimens were compared using miRNA microarrays. All patients had primary advanced ovarian HGSC (Supplementary Table S1). Five samples in SF tissues and five corresponding FFPE samples from the same patients were compared using a Pearson correlation test (Figure 1). Three SF normal ovarian tissue samples from patients treated for benign uterine disease were used as a control. There was a significant correlation between the expression of each miRNA in all samples (*P* < 0.001).

### Patient characteristics

miRNA expression profiles associated with recurrent ovarian HGSC were analyzed in eight patients. All patients were in an advanced disease stage and primary cyto-debulking operations were performed in all cases (Table 1). All patients received post-operative adjuvant chemotherapy (POAC) consisting of six courses of paclitaxel and carboplatin. Secondary cytoreductive operations were performed at recurrence in seven patients. One patient (P3) had palliative surgery immediately following POAC due to intestinal obstruction; a tumor sample was obtained during this surgery. Two patients (P1 and P4) had platinum-sensitive recurrence and five patients (P2, P5, P6, P7, and P8) had marginally platinum-sensitive recurrence (platinum-free interval: 6–12 months). The median number of chemotherapy lines before secondary surgery was 2, with a range of 1 to 4 chemotherapy lines. In four patients (P1, P2, P3, and P4), specimens from both primary cyto-debulking operations and secondary surgeries were stored as SF tissue, and specimens from the other patients (P5, P6, P7, and P8) were stored as FFPE tissue.

**Table 1:** Patient characteristics

Patient number	Tissue type	Stage	Grade	Date of primary surgery	Number of chemotherapy lines	Date of secondary surgery	Site of recurrent tumor	PFS	Recurrence type
P1	SF	IIIc	G3	2010-02-08	1	2011-12-01	Pelvic cavity	16	PS
P2	SF	IIIc	G2	2010-12-09	1	2011-12-16	Lymph node	8	PS
P3	SF	IIIc	G2	2011-11-23	1	2012-07-10	Abdominal wall	0	PR
P4	SF	IIIa	G3	2009-10-05	1	2012-05-16	Pelvic cavity	27	PS
P5	FFPE	IIIc	G3	2006-05-30	3	2009-12-02	Left psoas muscle	6	PS
P6	FFPE	IV	G3	2010-12-24	1	2011-12-06	Sub-hepatic mass	6	PS
P7	FFPE	IV	G2	2011-08-18	1	2012-08-23	Liver	7	PS
P8	FFPE	IIIc	G3	2010-04-01	1	2011-03-23	Pelvic cavity	8	PS

PFS, progression-free survival (between the last chemotherapy and the date of secondary surgery); SF, snap-frozen; FFPE, formalin-fixed, paraffin-embedded; PS, platinum-sensitive recurrence; PS, platinum-resistant recurrence.

### miRNA expression profiles associated with recurrence in advanced HGSC

**Figure 2:**
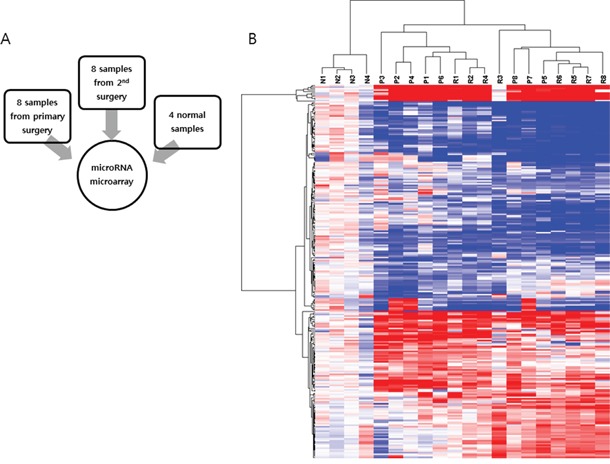
MicroRNA microarray analysis **A.** Eight pairs of primary-recurrent samples from eight patients were analyzed, and four normal ovarian tissue samples were used as a control. **B.** Significantly altered miRNAs are shown on a heatmap.

Eight pairs of primary-recurrent samples from eight patients were analyzed, with four normal ovarian tissue samples used as controls (Figure 2). miRNA expression profiles were compared between primary ovarian cancer tissue and normal ovarian tissue, recurrent ovarian cancer tissue and normal ovarian tissue, and recurrent ovarian cancer tissue and primary ovarian cancer tissue. In the comparison of primary ovarian HGSC with normal ovarian tissue, 57 miRNAs were significantly increased and 73 miRNAs were significantly decreased. These altered miRNA profiles are in agreement with previous findings [5]. In recurrent ovarian HGSC compared with normal ovarian tissue, 80 miRNAs were increased and 119 miRNAs were decreased. In recurrent ovarian HGSC compared with primary ovarian cancer tissue, 60 miRNAs, including *miR-630*, *miR-370*, and *miR-575*, were significantly increased and 52 miRNAs, including *miR-509-3p*, *miR-514a-3p*, and *miR-506-3p*, were decreased (Table 2). The top differentially expressed miRNAs (>2-fold change) in recurrent ovarian HGSC compared with primary HGSC are listed in Table 3. In addition, SF samples tended to cluster separately from FFPE samples, except for samples P3 and R3, in the heatmap (Figure 2).

**Table 2:** Numbers of significantly increased and decreased miRNAs identified using miRNA microarrays

	Significantly increased miRNAs	Significantly decreased miRNAs
Primary ovarian HGSC vs. normal ovarian tissue	57	73
Recurrent ovarian HGSC vs. normal ovarian tissue	80	119
Recurrent ovarian HGSC vs. primary ovarian HGSC	60	52

HGSC, high-grade serous carcinoma.

**Table 3:** Top differentially expressed miRNAs with >2-fold change in recurrent ovarian HGSC compared with primary ovarian tumor HGSC

Increased miRNAs	Fold change (log2 scale)	Decreased miRNAs	Fold change (log2 scale)
hsa-miR-630	2.48039	hsa-miR-509-3p	−3.6413
hsa-miR-370	2.09488	hsa-miR-514a-3p	−3.127
hsa-miR-575	2.08748	hsa-miR-506-3p	−2.4918
hsa-miR-3692-5p	1.72906	hsa-miR-508-3p	−2.3845
hsa-miR-4270	1.67729	hsa-miR-509-3-5p	−2.3436
hsa-miR-548q	1.67019	hsa-miR-95	−2.3274
hsa-miR-135a-3p	1.63426	hsa-miR-30a-3p	−2.2598
hsa-miR-1207-5p	1.62239	hsa-miR-362-5p	−2.0035
hsa-miR-4281	1.60895	hsa-miR-30b-5p	−2.0014
hsa-miR-671-5p	1.59902	hsa-miR-30e-3p	−1.9538

### Similar profiles of differentially expressed miRNAs in primary and recurrent ovarian HGSC compared with normal ovarian tissue

**Figure 3:**
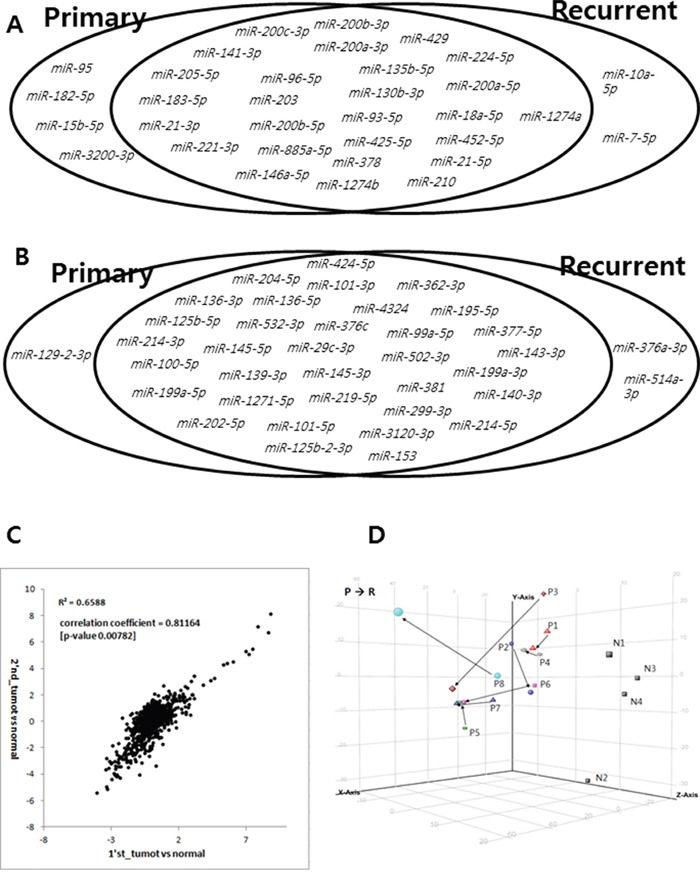
Characterization of miRNA expression **A.** Among 31 microRNAs upregulated by more than 4-fold in primary tumors, 27 were also significantly upregulated in recurrent tumor samples. **B.** Likewise, among 35 microRNAs downregulated by more than 4-fold in primary tumors, 34 were also significantly downregulated in recurrent tumor samples. **C.** Expression profiles of miRNAs in primary ovarian HGSC compared with those of normal ovarian tissue were similar to those in recurrent ovarian HGSC compared with normal ovarian tissue (correlation coefficient = 0.811, *P* = 0.0078). **D.** Primary and recurrent ovarian HGSC samples were not separated in three-dimensional principal components analysis, although normal ovarian specimens clustered together.

Among the 31 miRNAs that were increased by more than 4-fold in primary tumors, 27 were also significantly increased in recurrent tumor samples. Likewise, among the 35 miRNAs that were decreased by more than 4-fold in primary tumors, 34 were also significantly decreased in recurrent tumor samples (Figure 3, Supplementary Tables S2 and S3). Overall, the differentially expressed miRNAs identified in primary HGSC and recurrent HGSC compared with normal ovarian tissue were largely consistent. Specifically, *miR-200a*, *b*, *c*, *miR-141*, and *miR-429* were significantly increased in both primary and recurrent ovarian HGSC, and *miR-125*, *miR-99a*, and *miR-145* were significantly decreased in both primary and recurrent ovarian HGSC. The expression profiles of miRNAs in primary ovarian HGSC compared with normal ovarian tissue significantly correlated with those in recurrent ovarian HGSC compared with normal ovarian tissue (correlation coefficient = 0.81, *P* = 0.0078). In addition, primary and recurrent ovarian HGSC samples were not separated in three-dimensional principal component analysis, although the normal ovarian specimens clustered together.

### Validation of miRNA results

**Figure 4:**
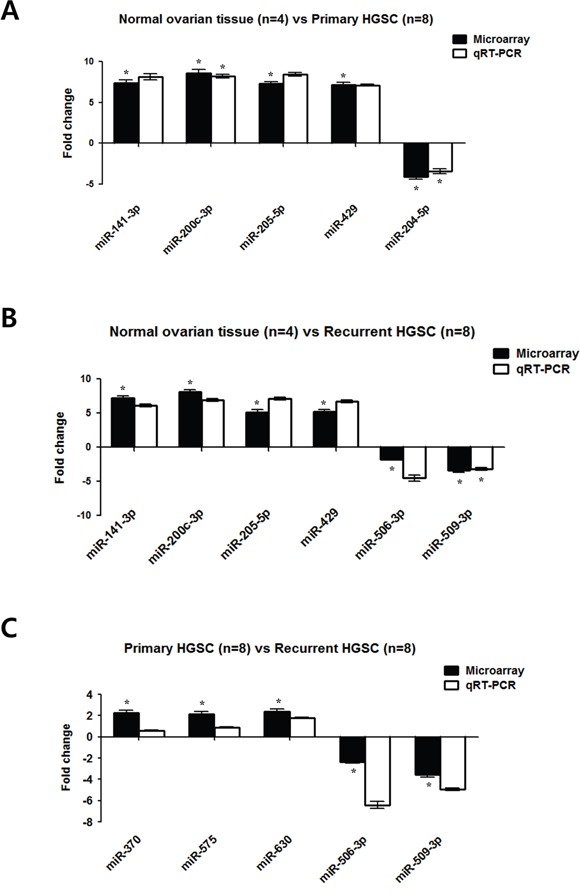
Real-time RT-PCR verification of the miRNA microarray results **A.** Differentially expressed miRNAs in primary ovarian HGSC (n = 8) compared with normal ovarian tissue (n = 4) from miRNA microarray experiments were consistent with data obtained using qRT-PCR. **B.** Differentially expressed miRNAs in recurrent ovarian HGSC (n = 8) compared with normal ovarian tissue (n = 4). **C.** Differentially expressed miRNAs in recurrent ovarian HGSC (n = 8) compared with primary HGSC (n = 8). Results are the mean ± standard deviation of three independent experiments performed in triplicate. (*P < 0.05).

Among the differentially expressed miRNAs, 5–6 were subjected to qRT-PCR analysis to validate the miRNA microarray results. We selected *miR-141*, *miR-200c*, *miR-205*, *miR-429*, *miR-204*, *miR-506*, and *miR-509-3p*, which were differentially expressed between normal ovarian tissue and ovarian HGSC (Figure 4). In agreement with the microarray results, the expression levels of *miR-141*, *miR-200c*, *miR-205*, and *miR-429* were increased, whereas those of *miR-204*, *miR-506*, and *miR-509-3p* were decreased in HGSC. We selected *miR-630*, *miR-370*, *miR-575*, *miR-506*, and *miR-509-3p*, which were differentially expressed between recurrent and primary ovarian HGSC. For the selected miRNAs, the *P*-value and FDR were calculated and were found to be significant (<0.05, Supplementary Table S4). In agreement with the microarray results, the expression levels of *miR-630*, *miR-370*, and *miR-575* were increased, whereas those of *miR-506* and *miR-509-3p* were decreased in the recurrent samples. Thus, the microarray data were validated, warranting further miRNA analyses in a clinical setting.

## DISCUSSION

In this study, we determined that the miRNA expression profiles in primary and recurrent ovarian HGSC were consistent (correlation coefficient = 0.81, *P* = 0.0078). Although some miRNAs were significantly increased or decreased in recurrent ovarian HGSC, most of the miRNAs matched with those of primary HGSC samples. To our knowledge, this is the first study to show a similarity of miRNA expression profiles between primary and recurrent ovarian HGSC.

A few previous studies explored miRNA expression profiles in recurrent ovarian HGSC compared with primary HGSC [10–13]. Laios *et al*. reported that *miR-9* and *miR-223* are potential biomarkers of recurrent epithelial ovarian cancer [11]. Chong *et al*. compared miRNA expression profiles in primary and recurrent ovarian serous carcinoma tissue [12]. However, primary and recurrent tumor specimens were not taken from the same patient in that study. Therefore, their resulting list of differentially expressed miRNAs was not consistent with our present data.

Recently, a whole-genome characterization of chemoresistant ovarian cancer tissue was published [13]. The authors showed that relapse samples had a higher mutational burden (single nucleotide variants and indels) at both the coding and non-coding levels than matched primary samples, suggesting that HGSC continues to evolve during treatment. In concordance with these findings, we found that recurrent tumors had a higher number of significantly increased or decreased miRNAs (Table 2).

The reason for the miRNA expression similarity between primary and recurrent HGSC has not yet been elucidated. We speculate that miRNAs are highly conserved throughout the species [14], and that miRNA profiles are not largely changed under the selective pressure of chemotherapy. Otherwise, HGSC may undergo clonal evolution under treatment, such that miRNA expression profiles of primary tumors could be conserved at recurrence.

Nevertheless, some miRNAs were significantly increased or decreased in recurrent HGSC compared with primary HGSC. The most significantly increased miRNA in recurrent HGSC observed was *miR-630*. Increased expression of *miR-630* in gastric cancer and colorectal cancer has been reported to be associated with poor survival [15, 16]. A proposed mechanism is that *miR-630* inhibits p53 activation by limiting the early DNA damage response [17]. *miR-630* was found to be increased in non-small cell lung cancer cells and head and neck squamous cell carcinoma cells in response to cisplatin treatment [18]. Upregulation of *miR-630* was also observed in epithelial ovarian cancer, and downregulation of *miR-630* using an inhibitor improved the chemosensitivity of ovarian cancer cells to cisplatin [19]. Thus, further functional validation of *miR-630* should be performed.

In the present study, we used different tissue types (SF and FFPE) for miRNA expression analyses. Previous miRNA expression studies have typically used FFPE specimens, and have shown that the miRNAs remain largely intact in routinely processed FFPE clinical specimens [20–22]. In agreement with previous studies, we also demonstrated strong correlations between expression profiles in matched SF and FFPE specimens. In addition, miRNA expression profiles obtained using SF + FFPE specimens in primary ovarian HGSC compared with normal ovarian tissue were consistent with previous results obtained using SF specimens used for miRNA analysis of primary ovarian HGSC [5]. However, the SF samples tended to cluster separately from FFPE samples, except for P3 and R3, in the heatmap (Figure 3). These findings indicate that pooling different tissue types together could affect the miRNA expression profiling results.

A limitation of our study is that we did not perform correlation analyses between miRNA expression profiles and clinicopathologic factors owing to the limited number of cases. With the exception of one patient, the patients included in this study had chemosensitive recurrence at secondary surgery. Secondary cyto-debulking operations are usually considered in only platinum-sensitive recurrent cases. In addition, recurrent HGSC specimens usually have extensive fibrosis. Although we used samples with a high tumor percentage, the presence of fibrosis may have led to confounding miRNA expression results.

In conclusion, primary and recurrent ovarian HGSC displayed similar miRNA expression patterns. Nevertheless, some altered miRNAs could be implicated in the recurrence of ovarian HGSC, and therefore further studies are required to determine the functional role of differentially expressed miRNAs in recurrent ovarian HGSC.

## MATERIALS AND METHODS

### Patients and study design

**Figure 5:**
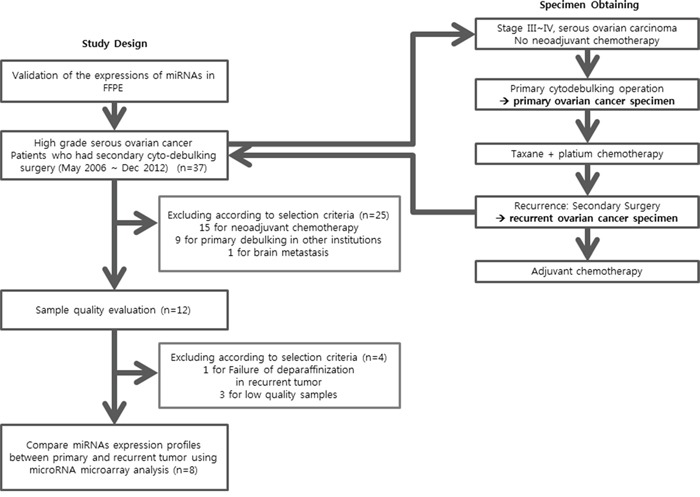
Study design and patient selection

The experimental protocol was approved by the Institutional Review Board of the Yonsei University College of Medicine (4-2012-0741). Between May 2006 and December 2012, 37 ovarian HGSC patients underwent secondary debulking surgery at recurrence (Figure 5). Among them, 15 patients received neo-adjuvant chemotherapy before primary surgery was excluded. Nine patients that underwent primary surgery at another institution were excluded because primary tumor tissues were not available. Finally, three patients were excluded because of the low quality of samples, and one patient was excluded owing to failure of deparaffinization. Therefore, for the final analysis, eight pairs of primary-recurrent tumor tissues from eight patients were used for miRNA microarray analysis. Snap-frozen fresh (SF) tissues stored in liquid nitrogen and archival formalin-fixed, paraffin-embedded (FFPE) tissues were used for miRNA expression analyses. This strategy was used because ovarian tumor samples with both primary and corresponding recurrent samples preserved as SF tissues were limited. Therefore, we initially validated the miRNA expression profiles of FFPE-preserved tissues compared with those of SF-preserved tissues. Four SF normal ovarian tissue samples from patients treated for benign uterine disease were used as a control; the age of the control patients ranged from 49 to 60 years. For primary HGSC, FFPE tissues were sampled at the point where the tumor percentage was above 90% based on hematoxylin and eosin (H&E) staining, and 10-μm serial sectioning was performed. In recurrent HGSC, the tumor density is relatively low and tumors are surrounded by stromal cells. Therefore, core tissue (2 mm) was obtained using a hollow needle at the point where the tumor percentage was above 90% based on H&E staining, as performed for the tissue microarrays.

### RNA preparation

Frozen tissue samples were homogenized in TRIzol® reagent (Invitrogen, Glasgow, UK) using an Omni-Mixer Homogenizer (Omni International, Kennesaw, GA, USA). Total RNA was then isolated according to the manufacturer's instructions. FFPE specimens were deparaffinized in xylene (tissue slides, 3 min; tissue core, overnight), and total RNA isolation was performed using a QIAamp RNA Mini Kit (Qiagen, Hilden, Germany). RNA quantity was assessed using a Nanodrop 1000A spectrophotometer (Nanodrop Technologies, Wilmington, DE, USA), and RNA quality was assessed using an Agilent 2100 Bioanalyzer and RNA 6000 Nano Chip (Agilent Technologies, Amstelveen, the Netherlands).

### miRNA microarray analysis

Samples of total RNA (100 ng) containing miRNA were labeled with cyanine 3-pGp (Cy3) using an Agilent miRNA Complete Labeling and Hyb Kit (Agilent Technologies, Santa Clara, CA, USA). Samples were placed on an Agilent Human miRNA v14 chip (AMDID 029297) and covered with a Gasket slide (Agilent Technologies, Santa Clara, CA, USA). Slides were hybridized for 16 h at 42°C using an Agilent hybridization system. miRNA arrays were analyzed using GeneSpring GX v11.5 (Agilent Technologies, Santa Clara, CA, USA) with standard normalization methods for one-channel microarrays with percentile median normalization methods. Fold-change values were calculated for unpaired comparisons between normal and test samples, and then averaged to generate a mean fold-change value. Welch's *t*-tests were used to determine statistical significance. Target predictions of significantly modified miRNAs were analyzed using the TargetScan 5.1 and miRBase v16 databases. miRNA microarray data from this study have been submitted to the Gene Expression Omnibus (GEO; http://www.ncbi.nlm.nih.gov/geo/info/geo_agil.html) under accession number GSE83693.

### Reverse transcription and quantitative real-time reverse transcription-polymerase chain reaction (qRT-PCR)

To validate results of the miRNA microarray experiments, the five most differentially expressed miRNAs were further evaluated using qRT-PCR. Total miRNA was isolated using a mirVANA microRNA Isolation Kit (Ambion, Austin, TX, USA) according to the manufacturer's protocol. Synthesis of cDNA was performed using TaqMan MicroRNA Assays (Applied Biosystems, Foster City, CA, USA). qRT-PCR was performed using an Applied Biosystems Prism 7500 Fast Sequence Detection System (Applied Biosystems, Warrington, UK) according to the manufacturer's protocol. Relative gene expression was analyzed using the 2^−ΔΔCT^ method, and results are expressed as the fold-change relative to control values. All experiments were repeated three times.

### Statistical analysis

To ensure the accuracy of microarray data analysis, false discovery rate (FDR)-adjusted *P*-values (q-values) were calculated using the methods reported by Storey [23]. The qRT-PCR data are expressed as the mean ± standard deviation of at least three independent experiments. Significance was determined using the Student's *t*-test. Comparison of miRNA expression profiles was performed using Pearson correlation. Statistical analyses were performed using SPSS (Version 18.0, SPSS, Inc., USA).

## SUPPLEMENTARY TABLES







## References

[1] Bookman MA (2013). Should studies of maintenance therapy be maintained in women with ovarian cancer?. J Gynecol Oncol.

[2] Jemal A, Siegel R, Xu J, Ward E (2010). Cancer statistics, 2010. CA Cancer J Clin.

[3] Hannon GJ (2002). RNA interference. Nature.

[4] Ambros V (2003). MicroRNA pathways in flies and worms: growth, death, fat, stress, and timing. Cell.

[5] Nam EJ, Yoon H, Kim SW, Kim H, Kim YT, Kim JH, Kim JW, Kim S (2008). MicroRNA expression profiles in serous ovarian carcinoma. Clin Cancer Res.

[6] Iorio MV, Visone R, Di Leva G, Donati V, Petrocca F, Casalini P, Taccioli C, Volinia S, Liu CG, Alder H, Calin GA, Menard S, Croce CM (2007). MicroRNA signatures in human ovarian cancer. Cancer Res.

[7] Cancer Genome Atlas Research N (2011). Integrated genomic analyses of ovarian carcinoma. Nature.

[8] Ross JS, Ali SM, Wang K, Palmer G, Yelensky R, Lipson D, Miller VA, Zajchowski D, Shawver LK, Stephens PJ (2013). Comprehensive genomic profiling of epithelial ovarian cancer by next generation sequencing-based diagnostic assay reveals new routes to targeted therapies. Gynecol Oncol.

[9] Boren T, Xiong Y, Hakam A, Wenham R, Apte S, Chan G, Kamath SG, Chen DT, Dressman H, Lancaster JM (2009). MicroRNAs and their target messenger RNAs associated with ovarian cancer response to chemotherapy. Gynecol Oncol.

[10] Gallagher MF, Heffron CC, Laios A, O'Toole SA, Ffrench B, Smyth PC, Flavin RJ, Elbaruni SA, Spillane CD, Martin CM, Sheils OM, O'Leary JJ (2012). Suppression of cancer stemness p21-regulating mRNA and microRNA signatures in recurrent ovarian cancer patient samples. J Ovarian Res.

[11] Laios A, O'Toole S, Flavin R, Martin C, Kelly L, Ring M, Finn SP, Barrett C, Loda M, Gleeson N, D'Arcy T, McGuinness E, Sheils O, Sheppard B, J OL (2008). Potential role of miR-9 and miR-223 in recurrent ovarian cancer. Mol Cancer.

[12] Chong GO, Jeon HS, Han HS, Son JW, Lee YH, Hong DG, Lee YS, Cho YL (2015). Differential MicroRNA Expression Profiles in Primary and Recurrent Epithelial Ovarian Cancer. Anticancer Res.

[13] Patch AM, Christie EL, Etemadmoghadam D, Garsed DW, George J, Fereday S, Nones K, Cowin P, Alsop K, Bailey PJ, Kassahn KS, Newell F, Quinn MC (2015). Whole-genome characterization of chemoresistant ovarian cancer. Nature.

[14] Voinnet O (2009). Origin, biogenesis, and activity of plant microRNAs. Cell.

[15] Chu D, Zhao Z, Li Y, Li J, Zheng J, Wang W, Zhao Q, Ji G (2014). Increased microRNA-630 expression in gastric cancer is associated with poor overall survival. PLoS One.

[16] Chu D, Zheng J, Li J, Li Y, Zhang J, Zhao Q, Wang W, Ji G (2014). MicroRNA-630 is a prognostic marker for patients with colorectal cancer. Tumour Biol.

[17] Galluzzi L, Morselli E, Vitale I, Kepp O, Senovilla L, Criollo A, Servant N, Paccard C, Hupe P, Robert T, Ripoche H, Lazar V, Harel-Bellan A (2010). miR-181a and miR-630 regulate cisplatin-induced cancer cell death. Cancer Res.

[18] Huang Y, Chuang A, Hao H, Talbot C, Sen T, Trink B, Sidransky D, Ratovitski E (2011). Phospho-DeltaNp63alpha is a key regulator of the cisplatin-induced microRNAome in cancer cells. Cell Death Differ.

[19] Zou YT, Gao JY, Wang HL, Wang Y, Wang H, Li PL (2015). Downregulation of microRNA-630 inhibits cell proliferation and invasion and enhances chemosensitivity in human ovarian carcinoma. Genet Mol Res.

[20] Hasemeier B, Christgen M, Kreipe H, Lehmann U (2008). Reliable microRNA profiling in routinely processed formalin-fixed paraffin-embedded breast cancer specimens using fluorescence labelled bead technology. BMC Biotechnol.

[21] Xi Y, Nakajima G, Gavin E, Morris CG, Kudo K, Hayashi K, Ju J (2007). Systematic analysis of microRNA expression of RNA extracted from fresh frozen and formalin-fixed paraffin-embedded samples. RNA.

[22] Zhang X, Chen J, Radcliffe T, Lebrun DP, Tron VA, Feilotter H (2008). An array-based analysis of microRNA expression comparing matched frozen and formalin-fixed paraffin-embedded human tissue samples. J Mol Diagn.

[23] Owzar K, Barry WT, Jung SH (2011). Statistical considerations for analysis of microarray experiments. Clin Transl Sci.

