# Supplementary material for: Primary and recurrent ovarian high-grade serous carcinomas display similar microRNA expression patterns relative to those of normal ovarian tissue
Source: Oncotarget. 2016 Sep 15;7(43):70524–34. doi: 10.18632/oncotarget.12045 (PMC5342571; doi:10.18632/oncotarget.12045)
Supplement: Supplementary file 1 [file oncotarget-07-70524-s001.pdf]

Primary and recurrent ovarian high-grade serous carcinomas display similar microRNA expression patterns relative to those of normal ovarian tissue

SUPPLEMENTARY TABLES

Supplementary Table S1: Characteristics of patients with primary ovarian HGSC

Patient Number	Stage	Grade	Date of primary surgery
AOC-a	IV	3	2011-1-17
AOC-b	IIIC	2	2010-3-29
AOC-c	IIIC	2	2009-6-1
AOC-d	IIIC	3	2010-2-15
AOC-e	IIIC	3	2009-8-11

Supplementary Table S2: Increased miRNAs in primary and recurrent ovarian HGSC compared with normal ovarian tissue

See Supplementary File 1

Supplementary Table S3. Decreased miRNAs in primary and recurrent ovarian HGSC compared with normal ovarian tissue

See Supplementary File 2

Supplementary Table S4: P-values and false discovery rates (FDRs) for specific miRNAs shown in Figure 5

**Normal ovarian tissue (n = 4) vs. primary HGSC (n = 8)**		
**Gene**	**P-value**	**q-value (FDR)**
miR-141-3p	0.000595	0.000991
miR-200c-3p	0.000367	0.000917
miR-204-5p	0.008053	0.008053
miR-205-5p	0.007306	0.008053
miR-429	0.000004	0.000021
**Normal ovarian tissue (n = 4) vs. recurrent HGSC (n = 8)**		
**Gene**	**P-value**	**q-value (FDR)**
miR-141-3p	0.001064	0.001595
miR-200c-3p	0.000821	0.002462
miR-205-5p	0.043884	0.043884
miR-429	0.002341	0.002809
miR-506-3p	0.000899	0.001595
miR-509-3p	0.000386	0.002318
**Primary HGSC (n = 8) vs. recurrent HGSC (n = 8)**		
**Gene**	**P-value**	**q-value (FDR)**
miR-370	0.009872	0.012340
miR-506-3p	0.019220	0.019220
miR-509-3p	0.006766	0.012744
miR-575	0.001498	0.007488
miR-630	0.007647	0.012340
