# Supplementary material for: Primary and recurrent ovarian high-grade serous carcinomas display similar microRNA expression patterns relative to those of normal ovarian tissue
Source: Oncotarget. 2016 Sep 15;7(43):70524–34. doi: 10.18632/oncotarget.12045 (PMC5342571; doi:10.18632/oncotarget.12045)
Supplement: Supplementary file 2 [file oncotarget-07-70524-s002.doc]

Supplementary Table 2. Increased miRNAs in primary and recurrent ovarian HGSC compared with normal ovarian tissue.

| Increased miRNAs in primary ovarian HGSC compared with normal ovarian tissue | Fold change (log2 scale) | Increased miRNAs in recurrent ovarian HGSC compared with normal ovarian tissue | Fold change (log2 scale) |
| --- | --- | --- | --- |
| hsa-miR-200c-3p | 8.81541 | hsa-miR-200c-3p | 8.14259 |
| hsa-miR-200b-3p | 8.60846 | hsa-miR-141-3p | 7.16443 |
| hsa-miR-141-3p | 7.84507 | hsa-miR-200b-3p | 6.70437 |
| hsa-miR-200a-3p | 7.43825 | hsa-miR-200a-3p | 5.46657 |
| hsa-miR-205-5p | 7.15762 | hsa-miR-429 | 5.28459 |
| hsa-miR-429 | 6.94852 | hsa-miR-205-5p | 5.07245 |
| hsa-miR-96-5p | 6.02251 | hsa-miR-96-5p | 4.49661 |
| hsa-miR-135b-5p | 5.54003 | hsa-miR-135b-5p | 4.38833 |
| hsa-miR-224-5p | 5.31541 | hsa-miR-224-5p | 4.29963 |
| hsa-miR-183-5p | 4.63187 | hsa-miR-21-3p | 3.43661 |
| hsa-miR-203 | 4.33837 | hsa-miR-183-5p | 3.28673 |
| hsa-miR-130b-3p | 3.76493 | hsa-miR-203 | 3.09945 |
| hsa-miR-200a-5p | 3.13566 | hsa-miR-10a-5p | 3.06586 |
| hsa-miR-21-3p | 3.09003 | hsa-miR-130b-3p | 2.76011 |
| hsa-miR-221-3p | 2.97981 | hsa-miR-1274a | 2.63182 |
| hsa-miR-200b-5p | 2.94678 | hsa-miR-885-5p | 2.53847 |
| hsa-miR-95 | 2.84731 | hsa-miR-1274b | 2.35449 |
| hsa-miR-93-5p | 2.80189 | hsa-miR-720 | 2.12031 |
| hsa-miR-18a-5p | 2.6709 | hsa-miR-210 | 2.08295 |
| hsa-miR-885-5p | 2.61655 | hsa-miR-221-3p | 2.04517 |
| hsa-miR-146a-5p | 2.53839 | hsa-miR-7-5p | 2.02212 |
| hsa-miR-182-5p | 2.52741 | hsa-miR-200b-5p | 2.01886 |
| hsa-miR-452-5p | 2.45824 | hsa-miR-196b-5p | 1.99251 |
| hsa-miR-1274a | 2.42343 | hsa-miR-602 | 1.94406 |
| hsa-miR-21-5p | 2.36734 | hsa-miR-1224-5p | 1.91668 |
| hsa-miR-378 | 2.30436 | hsa-miR-4286 | 1.89679 |
| hsa-miR-425-5p | 2.29994 | hsa-miR-522-3p | 1.88169 |
| hsa-miR-210 | 2.26642 | hsa-miR-21-5p | 1.8591 |
| hsa-miR-15b-5p | 2.22542 | hsa-miR-22-3p | 1.84041 |
| hsa-miR-3200-3p | 2.18599 | hsa-miR-3665 | 1.80752 |
| hsa-miR-1274b | 2.12103 | hsa-miR-378 | 1.78191 |
|  |  | hsa-miR-93-5p | 1.76294 |
|  |  | hsa-miR-378a-5p | 1.74864 |
|  |  | hsa-miR-521 | 1.70761 |
|  |  | hsa-miR-483-3p | 1.69561 |
|  |  | hsa-miR-1225-5p | 1.6454 |
|  |  | hsa-miR-1207-5p | 1.64386 |
|  |  | hsa-miR-4281 | 1.64164 |
|  |  | hsa-miR-425-5p | 1.61621 |
