# Supplementary material for: Primary and recurrent ovarian high-grade serous carcinomas display similar microRNA expression patterns relative to those of normal ovarian tissue
Source: Oncotarget. 2016 Sep 15;7(43):70524–34. doi: 10.18632/oncotarget.12045 (PMC5342571; doi:10.18632/oncotarget.12045)
Supplement: Supplementary file 3 [file oncotarget-07-70524-s003.doc]

Supplementary Table 3. Decreased miRNAs in primary and recurrent ovarian HGSC compared with normal ovarian tissue.

| Decreased miRNAs in primary ovarian HGSC compared with normal ovarian tissue | Fold change (log2 scale) | Decreased miRNAs in recurrent ovarian HGSC compared with normal ovarian tissue | Fold change (log2 scale) |
| --- | --- | --- | --- |
| hsa-miR-204-5p | -4.1174 | hsa-miR-204-5p | -5.3853 |
| hsa-miR-424-5p | -3.4337 | hsa-miR-362-3p | -5.1007 |
| hsa-miR-129-2-3p | -3.3847 | hsa-miR-101-3p | -4.953 |
| hsa-miR-101-3p | -3.3685 | hsa-miR-195-5p | -4.4016 |
| hsa-miR-362-3p | -3.3642 | hsa-miR-1271-5p | -4.2792 |
| hsa-miR-136-3p | -3.238 | hsa-miR-502-3p | -4.1663 |
| hsa-miR-136-5p | -3.1687 | hsa-miR-376a-3p | -4.1471 |
| hsa-miR-4324 | -3.0473 | hsa-miR-514a-3p | -4.05 |
| hsa-miR-195-5p | -3.0079 | hsa-miR-376c | -4.0319 |
| hsa-miR-125b-5p | -3.0068 | hsa-miR-29c-3p | -4.0131 |
| hsa-miR-532-3p | -2.8097 | hsa-miR-532-3p | -3.9046 |
| hsa-miR-376c | -2.7559 | hsa-miR-424-5p | -3.6242 |
| hsa-miR-99a-5p | -2.7459 | hsa-miR-377-3p | -3.5681 |
| hsa-miR-377-3p | -2.7412 | hsa-miR-136-3p | -3.5415 |
| hsa-miR-214-3p | -2.6489 | hsa-miR-660-5p | -3.4586 |
| hsa-miR-145-5p | -2.6145 | hsa-miR-219-5p | -3.4224 |
| hsa-miR-29c-3p | -2.5987 | hsa-miR-100-5p | -3.3868 |
| hsa-miR-502-3p | -2.5849 | hsa-miR-509-3p | -3.3501 |
| hsa-miR-143-3p | -2.5552 | hsa-miR-99a-5p | -3.3345 |
| hsa-miR-100-5p | -2.5029 | hsa-miR-4324 | -3.2099 |
| hsa-miR-139-3p | -2.4867 | hsa-miR-497-5p | -3.1469 |
| hsa-miR-145-3p | -2.4837 | hsa-miR-509-3-5p | -3.1345 |
| hsa-miR-381 | -2.4761 | hsa-miR-125b-5p | -3.1242 |
| hsa-miR-199a-3p | -2.4601 | hsa-miR-381 | -3.1015 |
| hsa-miR-199a-5p | -2.3859 | hsa-miR-143-3p | -3.0939 |
| hsa-miR-1271-5p | -2.3722 | hsa-miR-299-5p | -3.0515 |
| hsa-miR-219-5p | -2.3358 | hsa-miR-136-5p | -3.0399 |
| hsa-miR-299-3p | -2.2614 | hsa-miR-144-3p | -3.0311 |
| hsa-miR-140-3p | -2.2335 | hsa-miR-532-5p | -3.0118 |
| hsa-miR-202-5p | -2.0775 | hsa-miR-19b-1-5p | -2.9996 |
| hsa-miR-101-5p | -2.0619 | hsa-miR-502-5p | -2.9983 |
| hsa-miR-3120-3p | -2.0512 | hsa-miR-500a-3p | -2.9305 |
| hsa-miR-125b-2-3p | -2.0156 | hsa-miR-495 | -2.9289 |
| hsa-miR-153 | -2.0105 | hsa-miR-145-3p | -2.8549 |
| hsa-miR-214-5p | -2.0062 | hsa-miR-26b-5p | -2.8494 |
|  |  | hsa-miR-140-3p | -2.8458 |
|  |  | hsa-miR-3120-3p | -2.8187 |
|  |  | hsa-miR-129-2-3p | -2.8092 |
|  |  | hsa-miR-214-5p | -2.8073 |
|  |  | hsa-miR-140-5p | -2.6898 |
|  |  | hsa-miR-299-3p | -2.6675 |
|  |  | hsa-miR-199a-5p | -2.6584 |
|  |  | hsa-miR-29b-3p | -2.6389 |
|  |  | hsa-miR-508-3p | -2.6213 |
|  |  | hsa-miR-199a-3p | -2.6137 |
|  |  | hsa-miR-26a-5p | -2.5533 |
|  |  | hsa-miR-3117-3p | -2.5288 |
|  |  | hsa-miR-20a-3p | -2.5246 |
|  |  | hsa-miR-125b-2-3p | -2.5088 |
|  |  | hsa-miR-145-5p | -2.5006 |
|  |  | hsa-miR-10b-5p | -2.4689 |
|  |  | hsa-miR-34a-3p | -2.454 |
|  |  | hsa-miR-19a-3p | -2.4218 |
|  |  | hsa-miR-554 | -2.3952 |
|  |  | hsa-miR-30c-5p | -2.3925 |
|  |  | hsa-miR-101-5p | -2.3923 |
|  |  | hsa-miR-29c-5p | -2.3719 |
|  |  | hsa-miR-193a-3p | -2.3628 |
|  |  | hsa-miR-374a-5p | -2.3407 |
|  |  | hsa-miR-4319 | -2.2983 |
|  |  | hsa-miR-214-3p | -2.2627 |
|  |  | hsa-miR-153 | -2.2522 |
|  |  | hsa-miR-627 | -2.2463 |
|  |  | hsa-miR-30e-3p | -2.2384 |
|  |  | hsa-miR-29a-3p | -2.2314 |
|  |  | hsa-miR-362-5p | -2.1911 |
|  |  | hsa-miR-132-3p | -2.1909 |
|  |  | hsa-miR-19b-3p | -2.1664 |
|  |  | hsa-miR-411-5p | -2.1095 |
|  |  | hsa-miR-4328 | -2.109 |
|  |  | hsa-miR-664-3p | -2.1078 |
|  |  | hsa-miR-30b-5p | -2.0647 |
|  |  | hsa-miR-501-3p | -2.0576 |
|  |  | hsa-miR-450a-5p | -2.0379 |
|  |  | hsa-miR-202-5p | -1.991 |
